# Recent advances in host-focused molecular tools for investigating host-gut microbiome interactions

**DOI:** 10.3389/fmicb.2024.1335036

**Published:** 2024-03-28

**Authors:** Siyao Wang, Xu Gong, Fei Xiao, Yun Yang

**Affiliations:** ^1^Key Laboratory for Biomechanics and Mechanobiology of Ministry of Education, Beijing Advanced Innovation Centre for Biomedical Engineering, School of Engineering Medicine, Beihang University, Beijing, China; ^2^Key Laboratory of Big Data-Based Precision Medicine, Ministry of Industry and Information Technology, Beihang University, Beijing, China; ^3^Department of Thoracic Surgery, China-Japan Friendship Hospital, Beijing, China

**Keywords:** host-microbiome interactions, molecular tool, CRISPR, Cre-*loxP*, organoid 1

## Abstract

Microbial communities in the human gut play a significant role in regulating host gene expression, influencing a variety of biological processes. To understand the molecular mechanisms underlying host-microbe interactions, tools that can dissect signaling networks are required. In this review, we discuss recent advances in molecular tools used to study this interplay, with a focus on those that explore how the microbiome regulates host gene expression. These tools include CRISPR-based whole-body genetic tools for deciphering host-specific genes involved in the interaction process, Cre-*loxP* based tissue/cell-specific gene editing approaches, and *in vitro* models of host-derived organoids. Overall, the application of these molecular tools is revolutionizing our understanding of how host-microbiome interactions contribute to health and disease, paving the way for improved therapies and interventions that target microbial influences on the host.

## 1 Introduction

Gut microbiota is a community of indigenous microorganisms residing in the host intestinal tract, which has intimate interaction with the host and significantly regulate host physiology. Much efforts and significant advances have been made in understanding the relationship between human diseases and host-associated microbial communities, notably in the Integrative Human Microbiome Project [Integrative HMP (iHMP) Research Network Consortium, 2019]. Besides intestinal disorders (Imhann et al., 2018), gut microbial alterations were frequently observed in a wide range of diseases such as immune (Pellicciotta et al., 2019), neurological (Nikolova et al., 2021), and metabolic diseases (Yuan et al., 2022). Transplantation of dysregulated gut microbiome from diseased mice or patients has been observed to generally lead to similar syndromes in the recipient mice, indicating reciprocal interactions between the host and intestinal microbiome (Thaiss et al., 2016). Alterations in microbial consortia could be not only a consequence but also a cause in the pathogenesis of various diseases. Investigation of the mechanisms underlying host-microbiota interactions is crucial for promoting the understanding of the pathophysiology of microbiota-related diseases and the development of precise interventions.

Beyond the massive observational studies on the association between gut microbiome alterations and host diseases, further exploration on the causal link between the host-microbiota interaction in health and diseases has attracted much attention (Round and Palm, 2018; Koh and Bäckhed, 2020). On the basis of profile and function analysis of gut microbiota revealed by omics-based techniques, such as metagenomics, transcriptomics, proteomics and metabolomics, the causal role of these highly relative bacteria or bioactive microbial products in the host pathogenesis were further studied (Franzosa et al., 2015; Roager et al., 2019; Park et al., 2022). The causal role of these highly relative bacteria or bioactive microbial products in the host pathogenesis were further studied. Culturomics is an approach for extensive assessment of the microbial composition by high-throughput culture. With the aid of culturomics of gut microbiota as well as monocolonization of isolated bacterial strains in germ-free mice, critical gut bacteria which causally regulate host physiology have been illuminated (Round and Palm, 2018). To capture a spectrum of host-gut microbiota interaction scenarios, emerging molecular tools have been employed to delve into the underlying mechanisms within the host. The gene targets and signaling responses downstream gut microbiota are identified by genetically modified mice and host-derived organoid. The development and implementation of these molecular tools on the host have greatly advanced the deciphering of detailed molecular mechanism underlying host-microbiota interaction.

There have been some review articles summarizing microbiome-based approaches in studying host-microbe interaction (Round and Palm, 2018). In this review, we summarize a range of host-based molecular tools for deciphering the hypotheses about which genes or molecular are responded to microbial and how these factors modulate host phenotype and exploring the molecular mechanism underlying host-microbiome interaction. The host-targeting molecular tools including genome editing system, conditional gene manipulating system, tissue/cell specific gene delivery tools and platforms for simulating gut-host interaction are summarized, and their application in elucidating the host signaling response to gut microbiome are discussed. Then, we provided some considerations on how to select appropriate methods to be employed in complex host-microbial interactions. Then, we will illustrate the process of applying these strategies on elucidation the mechanisms of host-microbial interactions.

## 2 Whole-body genetic tools for deciphering host-specific genes involved in host-microbe interaction

### 2.1 CRISPR-Cas mediated whole-body gene manipulation

Based on the above studies, screening studies of microbial sequences can provide basis for how intestinal microorganisms cross-talk with the host. However, in the process of exploring specific mechanisms, there is still a lack of evidence of direct interaction. This requires researchers to further explore the genes responsible for host response. In addition to observational evidence, host gene knockouts are often performed *in vivo* to determine whether host-specific genes play a critical role in the response to microorganisms. Unveiling the host specific genes in response to gut microbiota is significant to understand the causal role of host-microbe interaction in host physiology and pathology. Remarkable alterations in transcriptional profiles of host tissues in response to gut dysbiosis have been reported in numerous studies, in relation to host physiology, including metabolism, immunity, intestinal barrier function (Schirmer et al., 2019), neurological regulation (Sharon et al., 2016), and tumor development (Gopalakrishnan et al., 2018). Based on transcriptomic analysis, researchers employ a diverse array of gene editing tools to further elucidate key host genes affected by the gut microbiota and deepen our understanding of their functions. Gene editing animal models have been instrumental in providing critical evidence demonstrating the involvement of host genes in host-microbe interactions in both health and disease contexts. These models are typically generated using genomic editing tools applied to embryonic stem (ES) cells. In contrast to earlier gene editing tools such as zinc finger nucleases (ZFNs) and transcription activator-like effector nucleases (TALENs), the emergence of clustered regularly interspaced short palindromic repeats and their associated Cas nucleases (CRISPR-Cas) has revolutionized the field. The CRISPR-Cas system offers several advantages, including simplicity of operation, high efficiency, and minimal off-target effects, allowing for the rapid generation of genetically modified animal models (Doudna and Charpentier, 2014).

**Figure 1:**
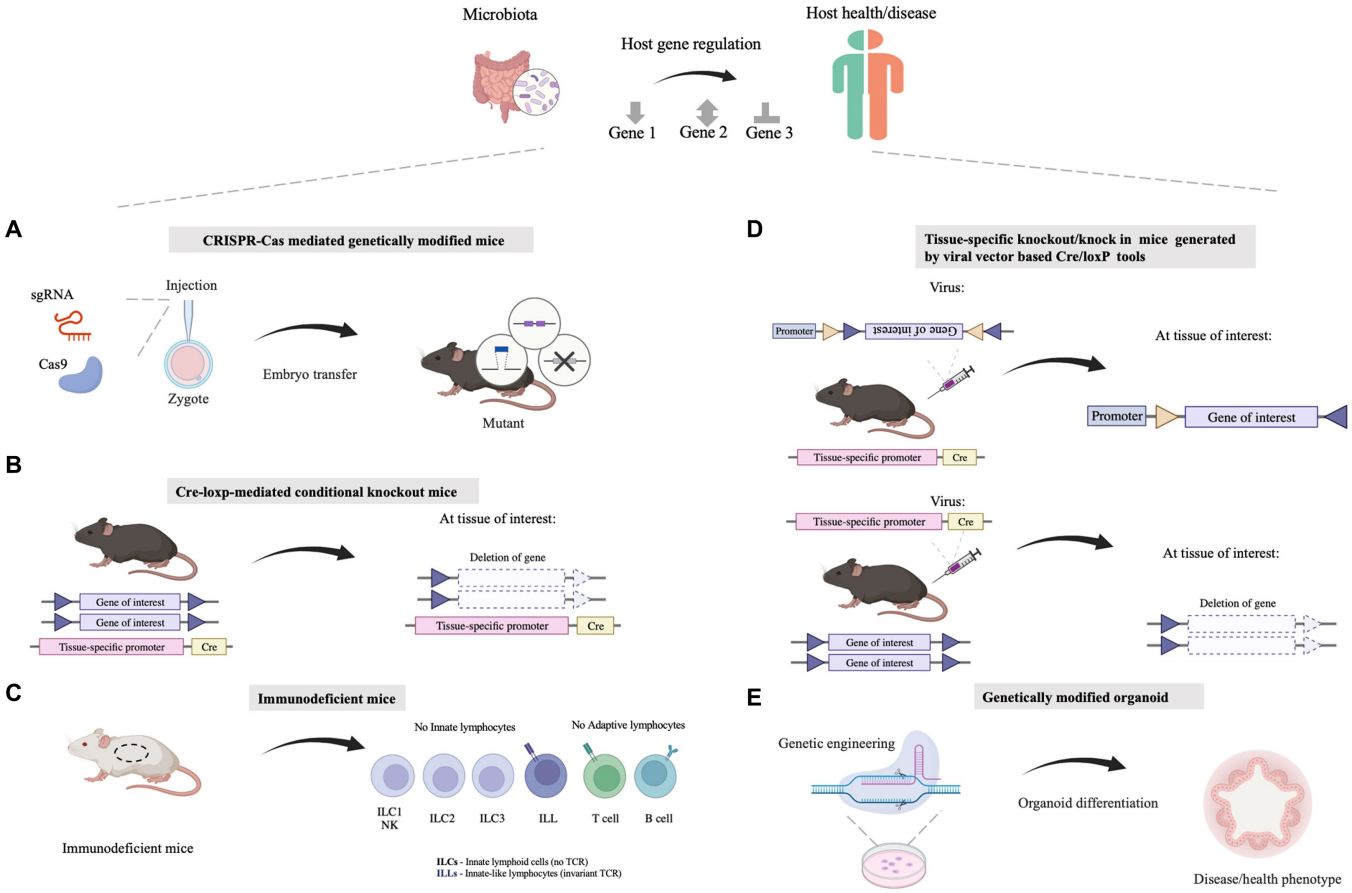
Molecular biology approaches facilitating the understanding of the mechanisms by which microbiota influences the host health. **(A)** The whole-body genetic tools are utilized for illustrating host-specific genes involved in host-microbe interaction. Tools like the CRISPR/Cas system are employed for genomic editing in mice. **(B)** Tissue/cell-specific genetically modified mice are exploited for illustrating the function of host genes in specific tissues/cells, which can explore the linkage between the gut and other organs/tissues as well. Tools like the Cre-*loxP* system are utilized for tissue/cell-specific genetic modification. **(C)** Immunodeficient mice which show defects in the innate immune system or (and) adaptive immune system are applied for dissecting the causality between the microbiome and the host immune system. **(D)** Viral vectors, such as adeno-associated viruses (AAVs) or lentivirus vectors, can be combined with existing Cre-*loxP* mouse lines together for Cre-*loxP*-mediated tissue/cell specific gene manipulation. **(E)** Genetically modified organoids are a powerful *ex vivo* model that deepens our insights into the mechanisms governing gene function and biological processes relating to host-microbe interaction. Illustrations are made with BioRender (biorender.com).

The CRISPR-Cas system was originally discovered as a prokaryotic adaptive immune defense system, being able to cut and destroy invading DNA in bacteria or archaea (Makarova et al., 2020). Following this discovery, the CRISPR-Cas system was heterologously introduced into eukaryotic cells to manipulate their genomes. When the CRISPR-Cas system is delivered into cells, guide RNA (gRNA) recruits the Cas enzyme to target a specific DNA sequence with a protospacer adjacent motif (PAM), and the Cas nuclease executes the gRNA-targeted cutting of DNA with a double-strand break (DSB) (Figure 1A). Various DNA manipulation strategies with Cas nuclease- or nuclease-deficient Cas have been applied to perform gene deletion, insertion, single-base conversion, and transcriptional regulation. DSB repair after Cas cleavage through the error-prone non-homologous end joining (NHEJ) pathway generates gene mutations, whereas DSB repair through homologous template-mediated recombination leads to fragment insertion into the DSB site (Kraft et al., 2015). Catalytically deficient Cas9 (dCas9) or Cas9 nickase (nCas9) fused to cytidine or adenosine deaminases can catalyze single-base conversion without generating DSBs (Li et al., 2017; Matsoukas, 2018). Moreover, the guidance of dCas9 to transcription-related elements could inhibit or activate the transcription of specific genes by regulating RNA polymerase binding (Gilbert et al., 2013; Qi et al., 2021).

These CRISPR-Cas mediated gene manipulation approaches have been implemented in experimental animals, in order to explore the molecular mechanism whereby the gut microbiota regulate host physiology (Doudna and Charpentier, 2014; Pickar-Oliver and Gersbach, 2019; Zhang, 2021). For several examples, inflammasomes are important sentinels and executors of innate immune defense, which play fundamental roles in regulating intestinal homeostasis and inflammatory reactions. NOD-like receptor (NLR) family members are inflammasome sensors for pathogen-associated molecular patterns (PAMPs) and damage-associated molecular patterns (DAMPs), which activate inflammasome assembly without the recognition of PAMPs or DAMPs, subsequently inducing pyroptosis and the release of proinflammatory cytokines (Xue et al., 2019). To study the influence of inflammasomes on host-microbiota interactions, mice with loss-of-function mutations in inflammasome components, including ASC and caspase-1, were generated using CRISPR-Cas tools. This series of mutant mice showed that inflammasones regulate intestinal innate immunity in response to gut commensal bacteria and their bioactive molecules (Rathinam and Chan, 2018). One such study reported that infection with pathogenic *E. coli* led to cell pyroptosis and increased secretion of proinflammatory cytokines, while ASC deletion repressed levels of cell pyroptosis and proinflammatory cytokines upon *E. coli* infection. Pre-treatment with the probiotic *L. rhamnosus* GR-1 protected the host against *E. coli* infection by suppressing pyroptosis, indicating that the host inflammasomes could be significantly regulated by pathogenic and probiotic bacteria (Wu et al., 2018). The CRISPR-Cas mediated gene manipulation approach also provides technical support for research on the impact of intestinal microorganisms on distal organs. In one example, IL-22 KO mice was utilized to verify the involvement of IL-22 expression in the process of dissecting the mechanism of fiber-mediated nourishment microbiota in gut affecting metabolic syndrome (Zou et al., 2018). In another example, IRF-1 and IRF-7 knockout mice were used to verify whether 18-hydroxyeicosapentaenoic acid produced by gut microbiota *Clostridium butyricum* promoted lung function through G protein-coupled receptor (GPR) 120 and IFN regulatory factor (IRF)-1/-7 activation (Hagihara et al., 2022).

CRISPR-Cas-mediated gene manipulation in experimental animals has facilitated the elucidation of the causal role of host genes in host-microbe interactions. Notably, some issues should be considered when using genetically engineered animals. First, the genetic manipulation of experimental animals may lead to a shift in gut microbiome profiles from those of wild-type animals. To rule out the influence of distant gut microbiota on the performance of genetically engineered animals, the intestinal microbial communities should be standardized among wild-type and mutant animals (Stappenbeck and Virgin, 2016). Second, it should be noted that the use of the CRISPR-Cas system for whole-body genetic manipulation of experimental animals is not suitable for essential genes, such as the *P53* gene (Maddalo et al., 2014), which are involved in regulating the cell cycle and DNA damage repair. There are also important tumor suppressor genes, such as the *PTEN* gene (Platt et al., 2014), whose knockout can lead to cancer and embryonic lethality. Notably, studies utilizing heterozygous animal models offer valuable insights into the physiological consequences of partial gene deficiency. For another instance, report indicates Igf1r^−/−^ mice experience perinatal lethality (Junnila et al., 2013). Mice heterozygous for the IGF-1R mutant allele (Igf1r^+/−^) were used to found they are typically around 10% smaller in size compared to wild-type counterparts. Additionally, investigations have shown that these mice display significant reductions in IGF-1-induced intracellular signaling, indicating a crucial role of IGF-1R in metabolic regulation (Holzenberger et al., 2003). This approach allows researchers to investigate the effects of gene dosage on various biological processes and provides a more nuanced understanding of gene function. Similarly, in the field of microbial flora research, mechanistic studies using partial gene deficiency can shed light on the intricate interactions between host and microbiota. Third, the behavior of mutant animals warrants additional analysis to ascertain whether the mutant gene triggers a robust compensatory mechanism, leading to the upregulation of redundant genes following knockout or knockdown. It is essential to perform certain common assays routinely employed (Table 1) in mutant animals to verify that any alterations in microbiome and host interactions are not attributable to the mutation itself. These tests encompass a range of behavioral, biochemical, and physiological parameters, allowing for a thorough assessment of the mutant phenotype and its potential impact on host physiology and microbiome composition.

**Table 1:** Typical behavioral and biochemical indicators to assess the gene-mutant mice.

Test type	Description	References
Body weight	Measure the body weight of mutant animals compared to wild-type controls	Speakman (2013)
Food consumption	Monitor the daily food intake of mutant animals to assess feeding behavior	Buettner et al. (2007)
Locomotor activity	Assess the spontaneous activity and movement patterns of mutant animals	Crawley (2007)
Cognitive function	Evaluate learning and memory using tasks like the Morris water maze or novel object recognition test	Vorhees and Williams (2006)
Blood chemistry	Analyze serum levels of glucose, cholesterol, triglycerides, and other metabolic markers	Shangguan et al. (2024)
Liver enzymes	Measure levels of enzymes such as alanine transaminase (ALT) and aspartate transaminase (AST) to assess liver function	Pratt and Kaplan (2000)
Intestinal permeability	Assess gut barrier function by measuring the passage of molecules from the gut into the bloodstream	Fasano (2012)
Gut microbiota analysis	Perform 16S rRNA sequencing or metagenomic analysis to characterize changes in the composition and diversity of the gut microbiome	Suzuki et al. (2019)

## 3 Tissue/cell-specific mutation for dissecting host-microbe interaction

Compared to gene manipulation in the whole body, gene-editing tools with higher resolution in cell types are preferred, especially for genes with housekeeping essentiality or tissue/cell-specific involvement in host-microbe interactions. Many studies have demonstrated the interaction between the gut microbiota and distal organs and tissues, such as the brain, liver, lung, and kidney, etc. Tissue/cell-specific molecular tools are necessary to decipher the molecular mechanisms underlying the gut-tissue/organ axis.

### 3.1 Cre-*loxP* based conditional gene modification

Cre-*loxP*-mediated conditional DNA recombination system makes it possible to study the role of tissue/cell-specific genes in host-microbiome interactions. The *Cre*-*loxP* system was initially isolated from P1 phage (Sternberg and Hamilton, 1981), and contains a recombinase enzyme Cre as well as DNA sequence named *loxP*. The *loxP* site is a 34 bp DNA sequence that is recognized by the recombinase enzyme Cre, and the recombinase Cre catalyzes homologous recombination between two *loxP* sites (Sauer and Henderson, 1988; McLellan et al., 2017). According to the arrangement of two *loxP* sites, gene deletion, inversion or translocation between two *loxP* sites can be induced (Bouabe and Okkenhaug, 2013; Kim et al., 2018). If two *loxP* sites are located on the same DNA strand in the same direction, Cre recombinase mediates the sequence deletion between the *loxP* sites. If two *loxP* sites are located on the same DNA strand in opposite directions, sequence inversion is catalyzed by recombination. When two *loxP* sites are arranged on different DNA strands or chromosomes, Cre recombinase triggers exchange or chromosomal translocation between the two DNA strands. In combination with the Cre-*loxP* system, tissue-specific promoters have been utilized to control location-programmable expression of the Cre enzyme and subsequent Cre-mediated tissue-specific gene manipulation (Figure 1B). A series of common promoters with tissue-specific expression properties is summarized in Table 2. In addition, engineered Cre recombinases with chemically inducible properties have also been applied to further achieve time-controllable gene modification in animal models to avoid abnormal early embryonic development or postnatal lethality (Kim et al., 2018). Spatiotemporally programmable gene modulation could be achieved with the Cre*-loxP* system equipped with both tissue/cell-specific promoters and chemically activated/inactivated Cre enzymes, including tamoxifen-inducible CreER and Dox (a tetracycline derivative) inducible Cre (Gossen and Bujard, 1992; Metzger and Chambon, 2001; McLellan et al., 2017).

**Table 2:** Summary of the cell type-specific or tissue-specific promoters to drive Cre expression.

Tissue/cell	Specific functional position	Targeted *Cre*/*CreERT2* promoter	References
Intestinal	Enteroendothelial cell	Cdh5	Zhang et al. (2018)
	Stem cells	Lgr5	Gronke et al. (2019)
	Epithelial cell	Villin	Thaiss et al. (2018), Cioppi et al. (2019), Grootjans et al. (2019), Reed et al. (2019), Kazgan et al. (2014)
Immune cell	Macrophage	Lysosome M (LC)	Bandaru et al. (2019)
		Lyz2	Larson-Casey et al. (2016)
		LysM	Wanderley et al. (2018)
	Dendritic cells	CD11c	Ainsua-Enrich et al. (2019), Mayer et al. (2017)
	Mast cells	CPA3	Wei et al. (2018), Chmelar et al. (2016)
	T-cell	CD4	Yoshida et al. (2019), Milam et al. (2018), Zeboudj et al. (2018)
		CD2	Vacchio et al. (2014)
	B-cell	Mb1	Wang et al. (2019), Forster et al. (2017)
		CD19	
		CD21	
Brain	Pan-GABAergic	Slc32a1	Daigle et al. (2018)
	Glutamatergic	Slc17a6	
		Slc17a8	
	Ventromedial hypothalamus	Fezf1	
	Claustrum	Gnb4	
	Thalamus	Tnnt1	
Liver	/	Albumin	Chao et al. (2018), Li M. et al. (2019)
Adipose tissue	/	Adipoq	Lee et al. (2019)
Musculoskeletal	Osteoclasts	Ctsk	Tonna et al. (2014)
Stomach	/	Tff1	Thiem et al. (2016)
Kidney	/	Gdnf	Cebrian et al. (2014)
	Nephron progenitor cells	Six2	Li S. Y. et al. (2019)
Pancreas	/	Gcg (Glu)	Shiota et al. (2017)
		Ins1 (MIP)	Thorens et al. (2015)
Skin	/	Krt14	Bhattacharya et al. (2018)
		K5	Yoon et al. (2019)

The Cre-*lox*P-mediated conditional gene editing avoids lethal mutations due to its controllable gene expression and enables the study of essential genes in host-microbe interactions (Cioppi et al., 2019; Gronke et al., 2019). Such conditional genetic manipulation has been applied to specific cell types in experimental animals, which has helped elucidate the effects of the microbiome on specific host tissues or cell types (Baier et al., 2020; Hinrichsen et al., 2021). For example, hexokinase 2 (HK2) is highly expressed in the gut epithelium and catalyzes the first and rate-limiting step of glycolysis. It plays a key role in normal embryonic development and adult organisms. HK2 knockout may lead to embryonic lethality or severe metabolic disorders. While, HK2 is upregulated in the epithelium of patients with colitis. To determine the impact of gut microbiota on epithelial HK2 in the pathogenesis of intestinal inflammation, researchers generated mice lacking the HK2 gene specifically in intestinal epithelial cells with the aid of the Cre-*loxP* system. Mice with a deletion of epithelial HK2 were less susceptible to acute colitis with reduced mitochondrial respiration and epithelial cell death. A probiotic microbe-derived metabolite, butyrate, repressed the expression of HK2 and protected wild-type, but not mutant, mice from colitis. These findings indicated that intestinal butyrate promotes intestinal hemostasis by repressing epithelial HK2 to attenuate intestinal inflammation (Hinrichsen et al., 2021).

Notably, conditional gene editing tools are powerful tools for investigating the molecular mechanism underlying the gut-organ/tissue axis, illustrating the interaction between the gut and distal organ/tissue (Malik et al., 2018; Saeedi et al., 2020). For instance, researchers have found that the administration of the probiotic bacterium *Lactobacillus rhamnosus* can protect mice against oxidative liver injury. To explore whether the therapeutic efficacy of *L. rhamosus* was dependent on the regulation of Nrf2-mediated antioxidant responses in the host liver, liver-specific Nrf2 knockout mice were constructed using the Cre-*loxP* system. The results showed that gut colonization by *L. rhamosus* protected the host against oxidative liver injury by stimulating Nrf2 in the liver, and the gut-liver axis was mediated by the microbial metabolite 5-methoxyindoleacetic acid of *L. rhamosus* (Saeedi et al., 2020).

### 3.2 Immunodeficient mice with specific immune cell populations

The intestinal mucus layer is located at the interface between the gut microbiome and enterocytes, separating commensal bacteria from the host epithelium, and the loose outer layer serves as the natural habitat for commensal bacteria. In addition to the epithelium and mucus layer serving as a physical barrier, the mucosal immune system plays a critical role in the defense against microbial threats. Diverse immune cells in the gut reside in the intestinal lamina propria and gut-associated lymphoid tissues, such as Peyer’s patches and mesenteric lymph nodes. The mucosal innate immune compartment contains cells of the mononuclear phagocyte system (MPS), e.g., as monocytes, macrophages, dendritic cells (DCs) (Zhao and Maynard, 2022), as well as Innate lymphoid cells (ILCs) (Guo et al., 2015) and natural killer cells (NK) (Yu et al., 2022). The adaptive immune systems in the gut contain T cells and plasma cells (Ivanov et al., 2022; Zhao and Maynard, 2022). Numerous studies have reported that gut microbes can significantly regulate host immunity, including innate immunity as well as adaptive immune responses in health and diseases (Lo et al., 2021). In order to explore the interaction between gut microbes and immune system, immunodeficient mice have been established as study tools (Figure 1C).

Immunodeficient mice were derived or bred from mutant mice with loss of function or impaired immune cell development. Mice deficient in innate immunity have been constructed, including beige mice (Song et al., 2017) (dysfunction of NK cell development by recessive mutations of *bg* gene induced by radiation) and ID2-deficient mice (Chen et al., 2015; Wang et al., 2017) (lacking all known ILC subsets). Immunodeficient mice with defects of adaptive immunity have been established, including Nude mice, XID mice, *rag*^−/−^ mice, SCID mice (Tsuchimoto et al., 2015), Foxp3^−^ DTR mice, etc. Nude mice (Chen et al., 2020; Lin et al., 2020) have defect of T cells induced by thymus excision. XID mice (Uslu et al., 2014) contain *xid* gene mutation with deficient B lymphocyte function and reduced antibody secretion. SCID mice (Thomsen et al., 2005) have a single recessive mutant gene in *scid* with congenital T and B cell defects. The *rag* genes are necessary for the maturation of T and B cells, while the *rag*^−/−^ mice possess deletion of *rag* genes and subsequent lack of mature T/B lymphocytes in peripheral blood (Koboziev et al., 2012; Wang et al., 2014; Atarashi et al., 2015). It has been reported that Foxp3^+^ Tregs were recognized as the major source of IL-10 expression, according to single cell RNA sequencing results. Hence, by means of tissue-specific intracellular expression of the diphtheria toxin fragment and supplement diphtheria toxin for 4–7 days, Foxp3-DTR mice were generated with deficiency in regulatory T cell (Treg) deficiency (Wang et al., 2014; Biton et al., 2018).

Immunodeficient mice have been applied to explore the host immune responses toward gut microbes. For example, Song et al. (2017) used T/B lymphocyte-deficient *Rag1*^−/−^ mice and natural killer (NK) cell-deficient Beige mice to study the mechanism underlying the antitumor effect of eustress stimulation. Exposure to enriched environment enhanced NK-cell activity against tumors and promoted tumoral infiltration of NK cells. Experiments with immunodeficient mice have shown that this effect remained intact in T/B lymphocyte-deficient *rag1^−/−^* mice, but was nearly eliminated in natural killer (NK) cell-deficient Beige mice or in antibody-mediated NK-cell-depleted mice, suggesting a predominant role of NK cells in enriched environment-induced tumor inhibition. In another work, *rag1*^−/−^ mice was employed to study the impact of segmented filamentous bacteria (SFB) on the host immunity. It was observed that SFB tightly adhered to the intestinal epithelium and induced Th17 cell differentiation in the intestine. While, *rag1*^−/−^ mice colonized with SFB-containing microbiota failed to induce chemokines including IL-17A, CXCL1 and CXCL2 and displayed defective neutrophil recruitment to the ileum, demonstrating that adaptive immunity is required for the IL-17A-mediated neutrophil recruitment in response to SFB colonization (Flannigan et al., 2017).

### 3.3 Tissue/cell specific gene delivery system

Tissue/cell specific gene delivery systems play a pivotal role in manipulating gene expression within targeted tissues or cell types in a labor and time-conserving manner. These delivery systems encompass both viral vectors and non-viral vehicles with genetic modifications to enhance tissue specificity, delivery efficiency and biosafety (Bulcha et al., 2021; Zu and Gao, 2021). Their cargo molecules includes DNA, mRNA, gene editing proteins or ribonucleoproteins, which mediate diverse gene manipulation performances including gene knockdown, knockout, or upregulation (Raguram et al., 2022). By administration of gene delivery system with tissue/cell specific affinity, cargo molecules controlled by tissue-specific promoters or just *in situ* injection, tissue/cell specific gene regulation can be accomplished.

*In situ* injection involves directly injecting gene delivery system into the target tissue of interest, such as brain (Landeck et al., 2021), muscles (Kenjo et al., 2021), and myocardium (Gabisonia et al., 2019). This method restricts dissemination of the vehicle to the local area, minimizing its impact on other tissues and organs. To achieve this, the target position for injection requires thick parenchymal tissues or relatively enclosed interstitial spaces, and the types of tissues suitable for *in situ* injection are limited. In addition, techniques such as stereotactic injection of the brain or intravitreal injection have a high level of technical complexity.

Delivery tools with tissue- or cell-specific affinities have been frequently utilized to generate programmable gene modifications. A classic gene delivery tool is a tissue-specific serotype of adeno-associated virus (AAV) vectors, and the delivery process occurs through the binding of cell surface receptors to AAV capsid proteins (Agbandje-McKenna and Kleinschmidt, 2011). The structures of capsid proteins exhibit varied affinities toward the cell surface receptors from distinct cells/tissues, thereby contributing to the cell/tissue tropism and determination of AAV serotypes (Agbandje-McKenna and Kleinschmidt, 2011). By modifying the capsid proteins of AAV, tissue-specific AAV serotypes have been obtained, thus enabling tissue-specific targeting and gene regulation (Büning and Srivastava, 2019). For instance, due to the efficient transduction of retinal neurons, AAV serotype 2 (AAV2) has been widely used to efficiently deliver transgenes to retinal ganglion cells (Lebherz et al., 2008). AAV2 has been clinically employed in Luxturna, the pioneering ocular AAV gene therapy that has gained approval in both the United States and Europe (Russell et al., 2017).

Another gene delivery tool is surface-modified non-viral vector for targeted gene delivery. For example, lipid nanoparticles, when coated with Apolipoprotein E (ApoE) lipoproteins, can effectively engage with liver cells via binding to their low-density lipoprotein (LDL) receptors (Paunovska et al., 2022). This interaction subsequently leads to the internalization of lipid nanoparticles by hepatocytes (Paunovska et al., 2022). Many studies have employed this lipid nanoparticle-based delivery system to effectively deliver Cas9 nuclease mRNA and single-guide RNAs (sgRNAs) to the liver for liver-specific gene editing (Raguram et al., 2022).

To precisely achieve tissue-specific gene modification, tissue- or cell-specific promoters (Table 2) has also been used to enhance the specificity of gene delivery. Typically, these promoters are used in conjunction with the Cre-*loxP* system as cargo for the delivery system by employing the following two strategies (Figure 1D). One way is to acquire gene knockout mice using *Cre*-expressing vectors and a mouse line whose gene of interest is flanked by *loxP* sites. The tissue/cell-specific gene knockout process can be accomplished with the help of vectors expressing *Cre* under the control of a tissue/cell-specific promoter after efficient *in vivo* vehicle delivery. Another strategy involves transfecting the virus vector, containing a gene editing agent flanked by two incompatible pairs of *loxP* sites, into transgenic mice with tissue/cell-specific expression of *Cre*to achieve double-floxed inverse orientation (DIO) or double-floxed orientation (DO) switches (Saunders et al., 2012). Catylyzed by *Cre* recombination, which is produced by certain tissues/cells in mice, the inversion of the target cassette and the excision of two *loxp* sites occur by two homologous recombination steps of *loxP* sites, finally leading to the expression or knockdown of the target gene in mice. In one study investigating how the iron levels of adipocytes influence fat absorption in the gut and obesity syndromes, adipocyte-specific iron levels were regulated by regulating the expression level of the iron exporter gene, *Fpn1* (Zhang et al., 2021). he researchers intraperitoneally injected Adipoq-Cre mice (JAX028020, an adipose-specific-Cre expression transgenic mouse line) with AAVs encoding Cre-dependent Fpn1C326S increase the expression of Fpn1 in adipose tissue (Zhang et al., 2021). The resultant AAV-Fpn1C326S mice showed a depleted iron level from mature adipocytes, and was more resistant to metabolic dysregulation induced by high-fat diet (Zhang et al., 2021).

## 4 *In vitro* models of host-derived organoid and gut-on-chip

### 4.1 Host-derived organoid

**Figure 2:**
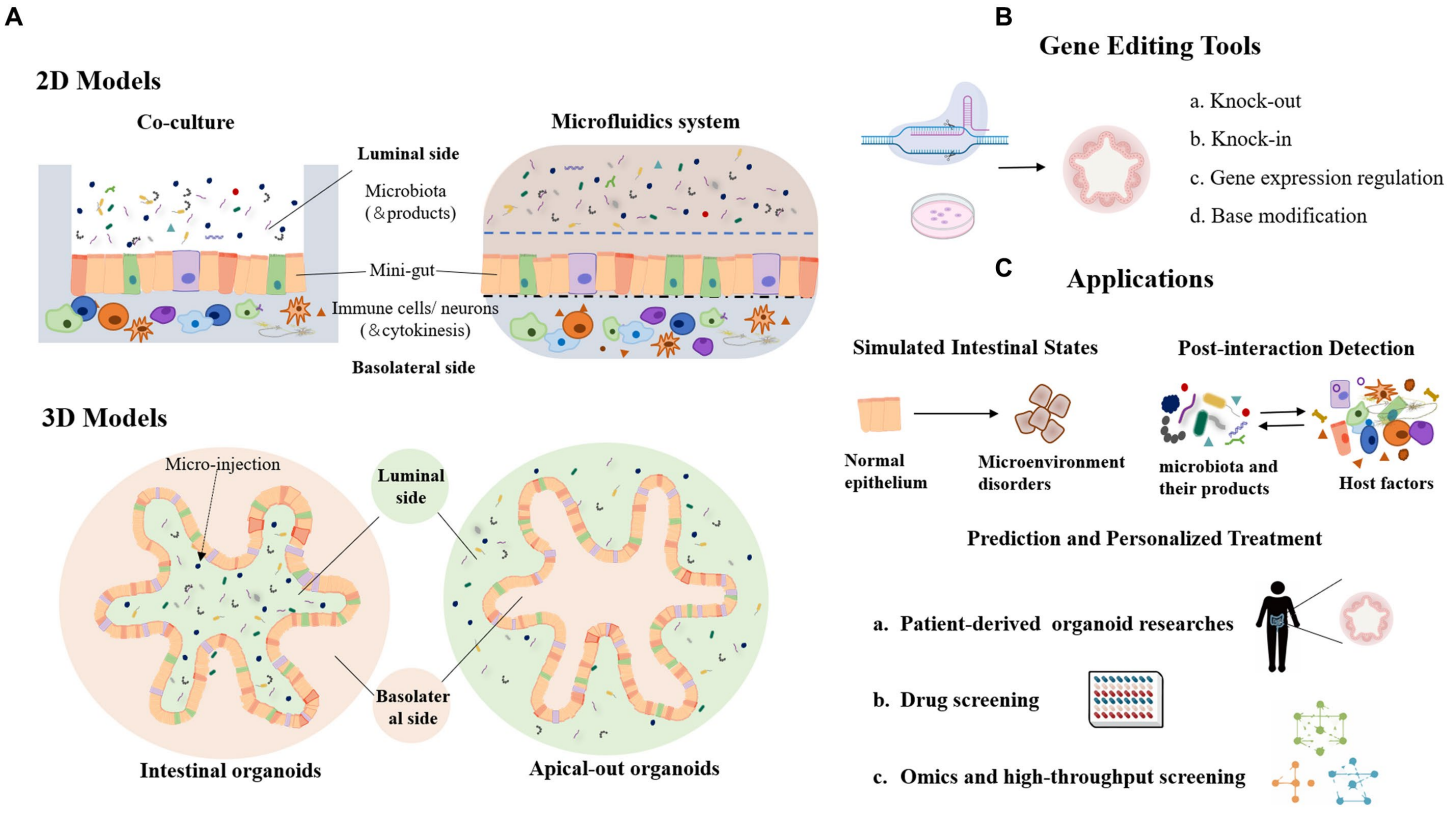
Organoid model and its application in the study of host-microbial interaction. **(A)** 2D and 3D organoid models in host-microbe interactions. **(B)** Genetic modification tools used in organoid models. **(C)** Lists of the main application scenarios of organoids.

The intestinal epithelium is a highly organized tissue with a crypt architecture and multiple subsets of epithelial cells (Helander and Fandriks, 2014). Many *in vitro* studies of host-microbe interactions have employed two-dimensional (2D) cell culture models to verify the influence of gut microbes on the host. However, 2D cell culture cannot resemble the architecture and function of the gut, which limits the findings of host-microbe interactions based on this culture platform. Recent advances in three-dimensional (3D) culture techniques have enabled the development of organoids (Figure 2A). Organoids are representative models mimicking the gastrointestinal system, with crypt-like structures and entire subsets of epithelial cells, providing *in vitro* platforms for molecular mechanism studies and bioactive compound screening (Mallott and Amato, 2021).

Organoids are generally formed by pluripotent stem cells (PSC) or organ-restricted adult stem cells (aSCs) (Barker et al., 2007; Sato et al., 2009). For example, adult intestinal stem cells expressing LGR5 can be cultured into a polarized intestinal epithelium with villus-like structures and crypt-like proliferative zones, and contain almost all intestinal epithelial cell types, including enterocytes, goblet cells, Paneth cells, and M cells (Sato et al., 2009). In the early development of organoids, the apical membrane of epithelial cells usually faces the inner lumen of the organoid, resulting in crucial transporters, cell surface receptors, and mucus layer located inside the organoid lumen. In order to study host-microbe interactions involving the apical polarity of epithelial cells, efforts have been made to reverse enteroid polarity, such that the apical surface everts to face the media (Figure 2A). These apical-out organoids maintain proper polarity and barrier function, differentiate into major epithelial cell types, and exhibit polarized absorption of nutrients (Co et al., 2019).

Organoids have been frequently applied as powerful *in vitro* platform for dissecting host-microbe interaction, due to their superior performance in facile treatment, modification and examination. For instance, the invasion mechanism of *S. typhimurium* was revealed by the organoids generated from human induced pluripotent stem cells. Then, imaging and sequencing were used to assess bacteria invasion and altered patterns of transcriptional expression after the exposure to bacteria (Forbester et al., 2015). In another study, organoids were used to explore a protective effect of the probiotic *L. acidophilus* against *S. typhimurium*-induced damage of intestinal epithelium (Lu et al., 2020).

Organoids can be propagated for a long time with stable genotype, and are amenable to many engineering techniques. Gene editing tools such as CRISPR-Cas9-based gene-editing systems have been widely used in organoids, generating gene knockout, insertion, expression regulation or single base modification (Figures 1E, 2B). For instance, CRISPR-Cas9 mediated modification of human stem cell-derived organs has been employed to study the origin of mutational markers of colorectal cancer (Drost et al., 2017). CRISPR-Cas9 technology was applied to human colon organoids to delete several potential DNA repair genes and mimic the deficiency in mismatch repair of colorectal cancer. Another study truncated the APC tumor suppressor gene in intestinal organoids using the CRISPR-Cas9 system, which resembled a well-recognized early event in the development of colorectal cancer (Sato et al., 2009). Moreover, gene delivery tools such as AAVs have also been used to make modification on mature organoids (Polyak et al., 2008; Vidovic et al., 2016). In the future, patient stem cell-derived organoids could be pathologically investigated in patient-specific settings and therapeutic targets screened from the perspective of host-microbial interactions (Spence et al., 2011; McCracken et al., 2014; Clevers, 2016; Fatehullah et al., 2016). In addition, organoids can be constructed repeatedly to facilitate multiple tests or high-throughput screening (Figure 2C). By manipulated cell-containing bio-ink to construct active structures, bio-3D printing technology could be used for organoid bioprinting, generating centimeter-scale tissues with self-organized characteristics of tubular intestinal epithelium with crypts and villi structures (Brassard et al., 2021). In summary, these advanced organoids, in combination with tools, provide a powerful platform for the zoomed-in study of microbial-epithelial cross-talk with insights into detailed molecular mechanisms and functionally involved cell subsets.

### 4.2 Gut-on-chip

Gut-on-chip technology, typically comprising a microfluidic chip with cells representing the intestinal epithelium and microbial ecosystems, can also replicate the intricate environment of the human intestine *in vitro*. By emulating the intestinal microenvironment, including the mucosal layer, peristalsis, and interactions between microorganisms and the intestinal epithelium, this technology facilitates the investigation of interactions between the intestinal microbiota and the host. To facilitating disease modeling and treatment research, as well as research on microbial composition and function, Gut-on-chip platforms can introduce specific factors relevant to intestinal diseases, such as bacteria or mediators, to study their interactions with the host. This aids in understanding disease pathogenesis mechanisms and identifying potential treatments. For instance, Kim et al. (2012) developed a “human intestinal chip” that simulated the intestinal microenvironment and introduced microbial communities, enabling it to replicate conditions found in the intestine, including physiological movement and flow. Jalili-Firoozinezhad et al. (2019) reported a complex human intestinal microbiome chip that mimics the anaerobic environment in the intestine and supports stable microbial growth. These studies jointly reveal the importance of Gut-on-chip technology in simulating the interaction between intestinal microorganisms and the host, and provide important tools and methods for related research and drug development.

## 5 Discussion and prospect

Recent studies have uncovered an interrelationship between the gut microbiota and the host response that extends beyond the digestive system. Microbial metabolites can act as signaling molecules to regulate host physiological processes such as glucose homeostasis, lipid metabolism, and neurobehavioral functions (de Vos et al., 2022; Bastings et al., 2023). Moreover, the gut microbiota may influence the efficacy and toxicity of drugs by modulating drug absorption, metabolism, and excretion (Savage, 2020). Hence, investigating the molecular mechanisms underlying host-microbe interaction is significant to advance our understanding of gut dysbiosis associated diseases and pave the way for promising interventions. Early research efforts focused on utilizing omics-based techniques to aid in the comprehension of the gut microbiota composition and its association with host health. Here, we highlight the importance of host-focused methods in illuminating the mechanisms underlying the causal relationships between the gut microbiota and the host. This review summarized the host-related *in vivo* platforms of genetic-level techniques and *in vitro* platforms of organoid or bionic system which are applicable to host-microbe interaction study, and discussed their implementation in exploring the detailed molecular mechanism of host responses regulated by gut microbiota. Complementing the techniques we summarized, the utilization of engineered microbes and bacteriophages provided an additional avenue to modulate the functional capacities and diversity of the gut microbiota, enabling researchers to manipulate the microbial community and understand its functional consequences (Inda et al., 2019; Tripathi et al., 2024).

With the rapid development of efficient gene editing systems, future research endeavors can capitalize on these advancements to manipulate multiple genes simultaneously (McCarty et al., 2020). This will greatly facilitate investigations into host gene pathways or networks involved in microbiota-host interactions. More *in vitro* tools for mechanism research, such as organoid culture and gut-on-a-chip, will accelerate the development and integration of other host-derived and microbiota-based technologies. These advancements hold promise for achieving a deeper understanding of the sophisticated signaling network of host-gut microbiota interactions, as well as the development of personalized disease therapies.

## Author contributions

SW: Writing – original draft. XG: Writing – original draft. FX: Writing – review & editing. YY: Writing – review & editing.
